# Bioinformatics analysis combined with molecular dynamics simulation validation to elucidate the potential molecular mechanisms of Jianshen Decoction for treatment of osteoporotic fracture

**DOI:** 10.1097/MD.0000000000033610

**Published:** 2023-04-21

**Authors:** Weinian Liu, Weijian Chen, Mengting Hu, Guangwei Wang, Yuanhao Hu, Qi He, Yidong Xu, Jun Tan, Haibin Wang, Liwei Huo

**Affiliations:** a Guangzhou Orthopedic Hospital, Guangzhou University of Chinese Medicine, Guangzhou, Guangdong, China; b The Fifth Clinical College, Guangzhou University of Chinese Medicine, Guangzhou, Guangdong, China; c The First Clinical College, Guangzhou University of Chinese Medicine, Guangzhou, Guangdong, China; d The Third Clinical College, Guangzhou University of Chinese Medicine, Guangzhou, Guangdong, China; e Science and Technology Innovation Center, Guangzhou University of Chinese Medicine, Guangzhou, Guangdong, China; f Guangdong Provincial People’s Hospital’s Nanhai Hospital, Foshan, Guangdong, China; g Department of Orthopaedics of the First Affiliated Hospital, Guangzhou University of Chinese Medicine, Guangzhou, Guangdong, China.

**Keywords:** bioinformatics, Jianshen decoction, molecular docking, molecular dynamics simulations, osteoporotic fracture

## Abstract

Osteoporotic fracture (OPF) is a prevalent skeletal disease in the middle-aged and elderly. In clinical practice, Jianshen Decoction (JSD) has been used to treat OPFs. However, the specific effective components and mechanisms of JSD on OPF have not been explored. Therefore, this study used bioinformatics analysis combined with molecular dynamics simulation validation to explore the molecular mechanism of JSD treatment of OPF. Public databases (TCMSP, Batman TCM) were used to find the effective active components and corresponding target proteins of JSD (screening conditions: OB ≥ 30%, drug-likeness ≥ 0.18, half-life ≥ 4). Differentially expressed genes (DEGs) related to OPF lesions were obtained based on the gene expression omnibus database (screening conditions: adjust *P* value < .01, | log_2_ FC | ≥ 1.0). The BisoGenet plug-in and the CytoNCA plug-in of Cytoscape were used to derive the potential core target proteins of JSD in the treatment of OPF. The JSD active ingredient target interaction network and the JSD-OPF target protein core network were constructed using the Cytoscape software. In addition, the R language Bioconductor package and clusterProfiler package were used to perform gene ontology (GO)/Kyoto Encylopedia Of Genes And Genome (KEGG) enrichment analysis on core genes to explain the biological functions and signal pathways of core proteins. Finally, molecular docking and molecular dynamics simulations were carried out through PyMOL, AutoDockTools 1.5.6, Vina, LeDock, Discovery Studio (DS) 2019, and other software to verify the binding ability of drug active ingredients and core target proteins. A total of 245 targets and 70 active components were identified. Through protein-protein interaction (PPI) network construction, 39 core targets were selected for further research. GO/KEGG enrichment analysis showed that the DNA-binding transcription factor binding, RNA polymerase II-specific DNA-binding transcription factor binding, MAPK signaling pathway, and ErbB signaling pathway were mainly involved. The results of molecular docking and molecular dynamics simulations supported the good interaction between MYC protein and Quercetin/Stigmasterol. In this study, bioinformatics, molecular docking, and molecular dynamics simulations were used for the first time to clarify the active components, molecular targets, and key biological pathways of JSD in the treatment of OPF, providing a theoretical basis for further research.

## 1. Introduction

In middle-aged and older persons, osteoporotic fracture (OPF) is a prevalent skeletal disease. It is a low-energy or nonviolent fracture, also known as a fragility fracture, and is the severe stage of osteoporosis (OP), marked by high morbidity, high disability, and death rates, as well as significant medical expenses.^[1]^ One OPF occurs every 3 seconds worldwide, 50% of women and 20% of men will have their first OPF after age 50, and 50% of patients who have already had one will likely have another one; the risk of re-fracturing an osteoporotic vertebral fracture in a woman is 4 times higher than the risk of a vertebral fracture that has not already broken.^[2–5]^ OPF of the hip and vertebrae shorten life expectancy and can result in death rates of up to 20% and rates of permanent impairment of up to 50% in people who are bedridden for extended periods.^[6,7]^ In China, the prevalence of OP in adults over 50 in 2018 was 19.2%, with 6.0% of males and 32.1% of women affected. A total of 32.0% of adults over 65 had OP, with 10.7% of males and 51.6% of women affected. In middle-aged and elderly women, OP is a severe issue.^[8]^ The essential tenets of treating OPFs are displacement, immobilization, functional training, and anti-OP therapy. The effectiveness of conventional anti-OP medications, however, is not particularly good. Bisphosphonates have drawbacks as the therapy of choice for OP, including a significant prevalence of fever and flu-like side effects.^[9]^ And estrogen has the risk of causing endometrial cancer, breast cancer, etc.^[10]^ Therefore, it is essential to pick a medication that can be used for a long time and is also secure, trustworthy, and affordable. Chinese Medicine therapy may alleviate symptoms, boost bone density, and enhance the quality of life while remaining safe, according to clinical practice, and it has its benefits and peculiarities.^[11,12]^

The Jianshen Decoction (JSD) is based on the theory that Chinese medicine warms the liver and kidneys and strengthens the muscles and bones. It is mainly used for the treatment of primary OP and OPF. The combination of JSD and Calcium Carbonate D3 Chewable Tablets (II), which has a better safety profile than alendronate tablets and Calcium Carbonate D3 Chewable Tablets (II), can increase serum E2, osteoprotegerin, and IGF-I levels, increase bone mineral density, and reduce pain in OP patients with kidney yang deficiency.^[13]^ Additionally, treating postmenopausal OP patients with JSD and zoledronic acid together can greatly increase their bone density, dramatically reduce their pain, stimulate estrogen production, and encourage bone growth.^[14]^ The combined osteoblast transplantation of JSD-containing serum into the fracture site is also discovered to significantly raise vascular endothelial growth factor mRNA expression.^[15]^ The studies mentioned above show that JSD is effective at treating OP and aiding in the healing of fractures, but their potential pharmacological mechanisms of action and pathway interactions remain unclear and require further research.

It is common practice in the drug development process to employ molecular docking, which is based on the idea of ligand-receptor interactions. A fine depiction of biomolecules is provided by molecular dynamics (MD) simulations, which explain the dynamic behavior of biomolecules at the atomic level.^[16,17]^

**Figure 1.**
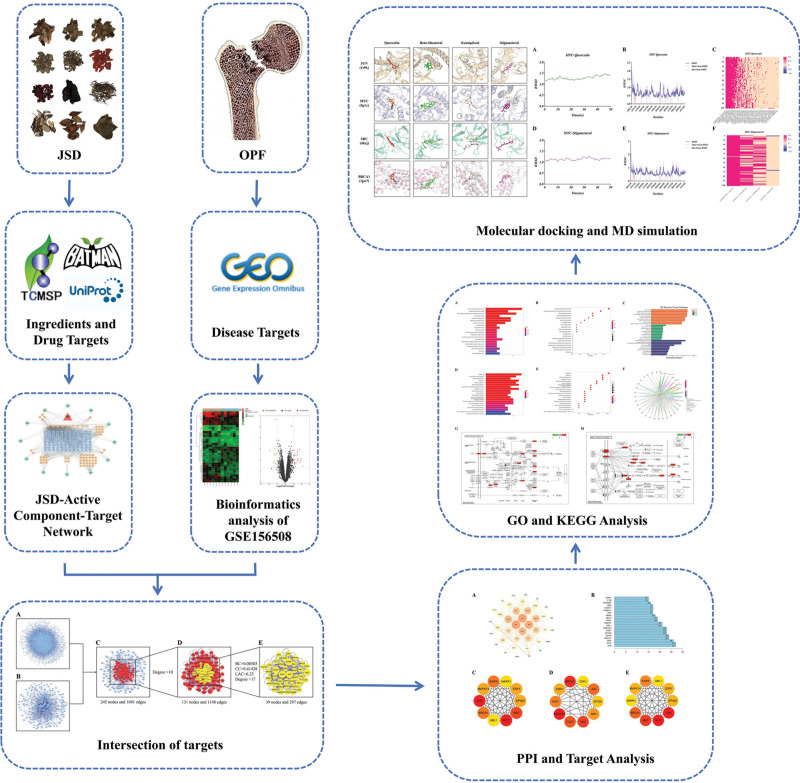
Workflow of this study.

We hypothesize that JSD has “multi-component, multi-target, and multi-pathway” characteristics for the treatment of OPF. This study aims to elucidate and validate the effective active ingredients, targets, and potential mechanisms of JSD in the treatment of OPF using bioinformatics, molecular docking techniques, and MD Simulation, and to provide a reference for subsequent basic research. The detailed workflow is shown in Figure 1.

## 2. Methods

### 2.1. Screening and identification of JSD effective active components

JSD is composed of 12 herbs: Eucommiae Cortex, Rhizoma Cibotii, Lycii Fructus, Rosae Laevigatae Fructus, Chaenomeles Sinensis (Thouin) Koehne, Achyranthis Bidentatae Radix, Flemingia Prostrata Roxb, Herba Taxilli, Cornus Officinalis Sieb. Et Zucc, Rehmanniae Radix Praeparata, Radix Clematidis, and Dipsaci Radix. The chemical composition of the above 12 herbs was searched using the TCMSP database (traditional Chinese medicine system Pharmacology, https://tcmsp-e.com/).^[18]^ Based on the search results, screening was performed according to ADME principles oral bioavailability ≥ 30%, Drug-likeness ≥ 0.18, Drug half-life ≥ 4),^[19–21]^ and the corresponding targets were obtained. For the unretrieved herb “Rhizoma Cibotii," the BATM-TCM database (http://bionet.ncpsb.org.cn/batman-tcm/)^[22]^ was used to retrieve the active ingredients with Change Score cutoff ≥ 20 and Adjusted *P* value cutoff ≥ .05, and the corresponding target sites were predicted. The UniProt database (https://www.uniprot.org) was used to import all of the target protein names identified throughout the screening, and target proteins without corresponding gene names were removed.

### 2.2. Acquisition and analysis of differentially expressed genes (DEGs) in bone tissue of OPF

High-throughput sequencing data of genes associated with OPF were downloaded from the gene expression omnibus database (https:/www.ncbi.nlm.nih.gov/geo/), and the screening conditions were: OPFs; human and whole genome sequencing data meeting the screening criteria were obtained. The microarray matrix file numbered GSE156508 and the GPL16686 microarray gene annotation file^[23]^ were obtained, which contained 12 femoral head samples, including 6 patients with OPF and 6 controls. FastQC software was used for quality assessment and quality control processing of the raw data, and the clean data obtained after quality control processing was further compared and analyzed using Hisat2 software to select the reference genome, and the HTSeq Python package was used for quantitative analysis of the number of gene reads to obtain the counts file. The collated counts data were filtered by Variance filter, Low abundance, and normalized using the Log_2_ Transformation. Differential analysis was performed using the DESeq2 package in R language to calculate the differential genes between the 2 groups, setting *P* < .05, adjusted *P* value < .01, and expression change greater than or equal to twofold (|log_2_FC|≥1.0) as the criteria for screening differential genes, where log_2_FC ≥ 1.0 represents gene expression up-regulation, log_2_FC ≤ −1.0 represents gene expression down-regulation, and finally the DEGs in the OPF group and the control group were derived, that is, DEGs for OPF. Heat map and cluster analysis of the screened differential genes were performed using the online analysis website ClustVis (https://biit.cs.ut.ee/clustvis/),^[24]^ and the *P* value of the processed data was -log_10_ transformed, and -log_10_ (*P* value) was grouped according to log_2_ FC for grouping (up-regulated gene group, down-regulated gene group, no statistical difference, and non-expression difference gene group), and import the processed data into bioinformatics tool to draw volcano map.

### 2.3. Screening of core target genes

The BisoGenet plug-in^[25]^ in Cytoscape 3.7.2 software^[26]^ was used to construct the protein-protein interaction (PPI) network of targets of JSD and the PPI network of DEGs of OPF, respectively. Using the “Networkanalyze” function^[27]^ in Cytoscape 3.7.2 software, the 2 PPI networks generated were intersected to obtain the PPI network shared by the targets of JSD and DEGs of OPF. The CytoNCA plug-in in Cytoscape 3.7.2 software was then used to calculate the multicenter network topology of the obtained shared PPIs and to filter them according to the median values of Degree, BetweennessCentrality , ClosenessCentrality, and local average connectivity values to derive the core target genes of active ingredients of JSD acting on OPF.

### 2.4. Intersection and interactive network construction of core target genes

The STRING (https://string-db.org/) online analysis website^[28]^ was applied to construct a potential core target gene interaction network for the core target genes by setting the protein species to “Homo sapiens,” settings minimum required interaction score to “medium confidence (0.400),” and hiding the unrelated targets. Count. R plug-in was applied to obtain the frequency of common protein targets. Further, the PPI network model of potential target genes was obtained by Cytoscape 3.7.2 software, and the cytoHubba plug-in was used to filter the hub genes, and the top 20 hub genes were ranked using the Degree algorithm, the PPI networks of the top 10 hub genes based on Degree, Betweenness, and Closeness were constructed separately.

### 2.5. Enrichment analysis of core target genes by gene ontology (GO)/Kyoto encylopedia of genes and genome (KEGG)

The GO analysis was performed separately for the core target genes by applying the clusterProfilerGO.R package in R language (https://www.r-project.org/) software.^[29]^ The KEGG Pathway enrichment analysis was performed with the clusterProfilerKEGG.R package, and the corresponding signaling pathways were mapped with the path view package. The core pathway enrichment was analyzed according to the enrichment factor values.

### 2.6. Molecular docking verification of the binding of the active component of the drug to the target protein

The structural formulas of the active ingredient were downloaded from the PubChem database (https://pubchem.ncbi.nlm.nih.gov/), and the corresponding 3D structures were made and exported to mol*2 format by Chem3D software, then the core protein were downloaded from the PDB database (http://www.rcsb.org/) structure domain in pdb format, and dehydration and dephosphorylation of the protein were performed using PyMOL software. AutoDockTools 1.5.6 software^[30]^ was used to convert the drug active ingredient and core protein gene files in pdb format to pdbqt format and to find the active pockets, and finally Vina script^[31]^ and LeDock software^[32]^ were run for molecular binding energy calculation and molecular docking results presentation, while Discovery Studio (DS) 2019 software to find docking sites and calculate the LibDockScore for flexible binding.^[33,34]^ The molecular docking results output from Vina software were imported into PyMOL software for molecular docking confirmation display. If the binding energy is <0, it means that the ligand and the receptor can bind spontaneously, and when the binding energy of Vina is ≤−5.0 kcal mol^−1^ and DS can find the docking site and LibDockScore ≥ 100, it means that the 2 forms stable docking, and the molecular docking results of ligand-receptor complexes are displayed in 3D and 2D to evaluate the bioinformatics Reliability of the predictions of the analysis.^[35]^

### 2.7. Molecular dynamics simulation of protein-ligand complexes

The force field parameters were obtained using the Simulation and Standard Dynamics Cascade modules of DS 2019 software, and the Charm force field was used for both the molecular parameters of the ligand and the molecular parameters of the receptor protein during the simulation, and the protein-ligand complex was solventized during the Solvation module calculation. Then we ran the molecular dynamics simulation, including 5 stages: I Minimization, I Minimization2, Heating, Equilibration, and Production. The system temperature was increased from 50 K to 300 K at 50 ns of simulated sampling with a time step set to 0.5 ns. The process was performed on an Normal Pressure and Temperature system and the temperature was set at a constant temperature of 300 K. Once the molecular dynamics calculations were completed, the Analyze Trajectory module was used to analyze the structural properties of the molecular dynamics trajectories, the number of non-bonded interactions per simulation frame, root mean square deviations (RMSD) and root mean square fluctuations between different conformations, and to detect non-bonded interactions formed between peptides and proteins.

## 3. Results

### 3.1. Effective active components of JSD

**Figure 2.**
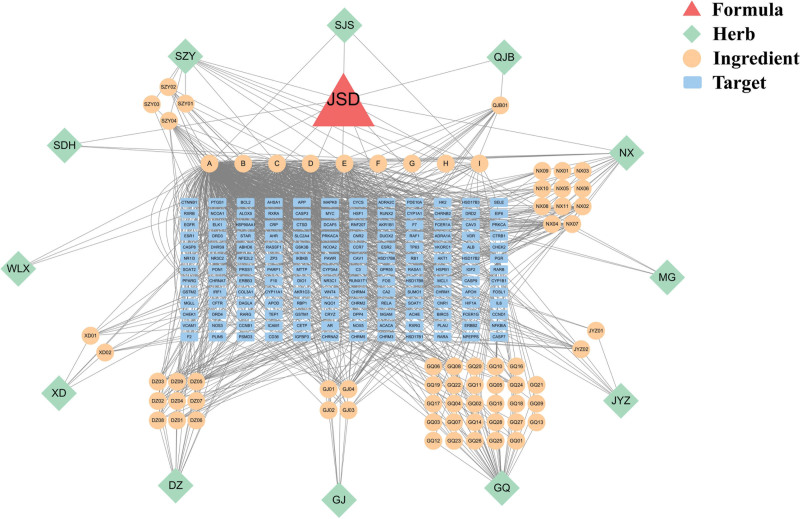
JSD-Active Component-Target Network. JSD = Jianshen Decoction.

A total of 70 active ingredients of JSD were searched and screened through the TCMSP database and BATMAN-TCM database, including 13 species of Eucommiae Cortex(*Eucommia ulmoides* Oliv.), 5 species of Rhizoma Cibotii[*Cibotium barometz(L.) J.Sm.*], 33 species of Lycii Fructus(*Lycium chinense* Mill.), 6 species of Rosae Laevigatae Fructus(*Rosa laevigata* Michx.), 3 species of Chaenomeles Sinensis (Thouin) Koehne[*Pseudocydonia sinensis* (Thouin) C. K. Schneid.], 15 species of Achyranthis Bidentatae Radix(*Achyranthes bidentata* Bl.), 1 specie of Flemingia Prostrata Roxb(*Flemingia prostrate* Roxb. f. ex Roxb.), 2 species of Herba Taxilli[*Taxillus sutchuenensis* (Lecomte) Danser], 10 species of Cornus Officinalis Sieb. Et Zucc(*Cornus officinalis* Sieb. et Zucc.), 2 species of Rehmanniae Radix Praeparata[*Rehmannia glutinosa* (Gaert.) Libosch. ex Fisch. et Mey.], 2 species of Radix Clematidis(*Clematis chinensis Osbeck*), and 4 species of Dipsaci Radix(*Dipsacus asper* Wall. ex Henry), and there were 9 ingredients common to multiple herbs (Table 1). 156 potential targets were obtained, and the Cytoscape 3.7.2 software was used to construct the JSD-Active Component-Target Network (Fig. 2), which obtained 239 nodes and 1252 edges; by analyzing the Degree values of active ingredient nodes and target gene nodes in the network, it was found that the top 5 active molecules in the comprehensive ranking were Quercetin, Beta-sitosterol, Kaempferol, Stigmasterol, Naringenin. And the top 5 targets were NCOA2, PGR, PTGS1, HSP90AA1, and PRKACA (Table 2).

**Table 1:** JSD effective active component information.

ID	MolId	Component	OB(%)	DL	HL	Herbs (Latin name)
DZ01	MOL000443	Erythraline	49.18	0.55	11.11	Eucommiae Cortex
DZ02	MOL002773	beta-carotene	37.18	0.58	4.36	
DZ03	MOL007059	3-beta-Hydroxymethyllenetanshiquinone	32.16	0.41	22.51	
DZ04	MOL008240	(E)-3-[4-[(1R,2R)-2-hydroxy-2-(4-hydroxy-3-methoxy-phenyl)-1-methylol-ethoxy]-3-methoxy-phenyl]acrolein	56.32	0.36	4.18	
DZ05	MOL009015	(-)-Tabernemontanine	58.67	0.61	19.73	
DZ06	MOL009027	Cyclopamine	55.42	0.82	14.67	
DZ07	MOL009029	Dehydrodiconiferyl alcohol 4,gamma’-di-O-beta-D-glucopyanoside_qt	51.44	0.40	7.54	
GJ01	-	Cudraphenone D	-	-	-	Rhizoma Cibotii
GJ02	-	Cudraphenone A	-	-	-	
GJ03	-	Bergapten	-	-	-	
GJ04	-	Naringenin	-	-	-	
GQ01	MOL000953	CLR	37.87	0.68	4.52	Lycii Fructus
GQ02	MOL001323	Sitosterol alpha1	43.28	0.78	5.64	
GQ03	MOL001979	LAN	42.12	0.75	5.84	
GQ04	MOL003578	Cycloartenol	38.69	0.78	5.00	
GQ05	MOL005438	campesterol	37.58	0.71	4.83	
GQ06	MOL007449	24-methylidenelophenol	44.19	0.75	5.10	
GQ07	MOL008173	daucosterol_qt	36.91	0.75	5.60	
GQ08	MOL008400	glycitein	50.48	0.24	16.32	
GQ09	MOL009604	14b-pregnane	34.78	0.34	4.57	
GQ10	MOL009617	24-ethylcholest-22-enol	37.09	0.75	5.32	
GQ11	MOL009618	24-ethylcholesta-5,22-dienol	43.83	0.76	5.76	
GQ12	MOL009620	24-methyl-31-norlanost-9(11)-enol	38.00	0.75	5.49	
GQ13	MOL009621	24-methylenelanost-8-enol	42.37	0.77	5.43	
GQ14	MOL009622	Fucosterol	43.78	0.76	5.44	
GQ15	MOL009633	31-norlanost-9(11)-enol	38.35	0.72	5.37	
GQ16	MOL009634	31-norlanosterol	42.20	0.73	5.27	
GQ17	MOL009635	4,24-methyllophenol	37.83	0.75	4.90	
GQ18	MOL009639	Lophenol	38.13	0.71	4.76	
GQ19	MOL009640	4alpha,14alpha,24-trimethylcholesta-8,24-dienol	38.91	0.76	6.67	
GQ20	MOL009641	4alpha,24-dimethylcholesta-7,24-dienol	42.65	0.75	5.44	
GQ21	MOL009642	4alpha-methyl-24-ethylcholesta-7,24-dienol	42.30	0.78	5.83	
GQ22	MOL009644	6-Fluoroindole-7-Dehydrocholesterol	43.73	0.72	5.10	
GQ23	MOL009646	7-O-Methylluteolin-6-C-beta-glucoside_qt	40.77	0.30	14.10	
GQ24	MOL009650	Atropine	42.16	0.19	5.27	
GQ25	MOL009656	(E,E)-1-ethyl octadeca-3,13-dienoate	42.00	0.19	5.47	
GQ26	MOL009677	lanost-8-en-3beta-ol	34.23	0.74	5.48	
GQ27	MOL009678	lanost-8-enol	34.23	0.74	6.41	
GQ28	MOL009681	Obtusifoliol	42.55	0.76	5.91	
JYZ01	MOL005030	gondoic acid	30.70	0.20	4.79	Rosae Laevigatae Fructus
JYZ02	MOL008628	4’-Methyl-N-methylcoclaurine	53.43	0.26	4.34	
NX01	MOL000085	beta-daucosterol_qt	36.91	0.75	5.02	Achyranthis Bidentatae Radix
NX02	MOL000173	wogonin	30.68	0.23	17.75	
NX03	MOL001006	poriferasta-7,22E-dien-3beta-ol	42.98	0.76	5.48	
NX04	MOL001454	berberine	36.86	0.78	6.57	
NX05	MOL001458	coptisine	30.67	0.86	9.33	
NX06	MOL002643	delta 7-stigmastenol	37.42	0.75	5.27	
NX07	MOL002714	baicalein	33.52	0.21	16.25	
NX08	MOL002776	Baicalin	40.12	0.75	17.36	
NX09	MOL002897	epiberberine	43.09	0.78	6.10	
NX10	MOL003847	Inophyllum E	38.81	0.85	15.51	
NX11	MOL004355	Spinasterol	42.98	0.76	5.32	
QJB01	MOL000665	flemiphilippinin C	47.66	0.73	15.01	Flemingia Prostrata Roxb
SZY01	MOL001771	poriferast-5-en-3beta-ol	36.91	0.75	5.07	Cornus Officinalis Sieb. Et Zucc.
SZY02	MOL005530	Hydroxygenkwanin	36.47	0.27	15.22	
SZY03	MOL005531	Telocinobufagin	69.99	0.79	5.15	
SZY04	MOL008457	Tetrahydroalstonine	32.42	0.81	10.55	
XD01	MOL003152	Gentisin	64.06	0.21	13.71	Dipsaci Radix
XD02	MOL009323	Sylvestroside III_qt	56.47	0.43	9.61	
A	MOL000098	quercetin	46.43	0.28	14.40	Eucommiae Cortex/Lycii Fructus/Rosae Laevigatae Fructus/Chaenomeles Sinensis (Thouin) Koehne/Achyranthis Bidentatae Radix/Herba Taxilli
B	MOL000211	Mairin	55.38	0.78	8.87	Eucommiae Cortex/Chaenomeles Sinensis (Thouin) Koehne
C	MOL000358	beta-sitosterol	36.91	0.75	5.36	Eucommiae Cortex/Lycii Fructus/Rosae Laevigatae Fructus/Achyranthis Bidentatae Radix/Cornus Officinalis Sieb. Et Zucc./Radix Clematidis/Dipsaci Radix
D	MOL000422	kaempferol	41.88	0.24	14.74	Eucommiae Cortex/Rhizoma Cibotii/Rosae Laevigatae Fructus/Achyranthis Bidentatae Radix
E	MOL000449	Stigmasterol	43.83	0.76	5.57	Lycii Fructus/Achyranthis Bidentatae Radix/Cornus Officinalis Sieb. Et Zucc./Rehmanniae Radix Praeparata/Radix Clematidis
F	MOL001494	Mandenol	42.00	0.19	5.39	Lycii Fructus/Rosae Laevigatae Fructus/Cornus Officinalis Sieb. Et Zucc.
G	MOL001495	Ethyl linolenate	46.10	0.20	6.20	Lycii Fructus/Cornus Officinalis Sieb. Et Zucc.
H	MOL002883	Ethyl oleate (NF)	32.40	0.19	4.85	Chaenomeles Sinensis (Thouin) Koehne/Cornus Officinalis Sieb. Et Zucc.
I	MOL000359	Sitosterol	36.91	0.75	5.37	Herba Taxilli/Cornus Officinalis Sieb. Et Zucc./Rehmanniae Radix Praeparata/Dipsaci Radix

DL = drug-likeness, HL = half-life, JSD = Jianshen decoction, OB = oral bioavailability.

**Table 2:** Degree, BetweennessCentrality and ClosenessCentrality values of the top 10 effective active components and targets of JSD.

Type	Description	Degree	BC	CC	Link	Rank
Component	quercetin	468	0.36587918	0.41608392	468	1
	beta-sitosterol	133	0.04782368	0.34795322	133	2
	kaempferol	116	0.23326832	0.42348754	116	3
	Stigmasterol	75	0.03554729	0.33615819	75	4
	Naringenin	28	0.18206499	0.30050505	28	5
	baicalein	22	0.04804266	0.32782369	22	6
	wogonin	22	0.04904434	0.34897361	22	7
	(-)-Tabernemontanine	17	0.02740133	0.33240223	17	8
	Tetrahydroalstonine	16	0.00894156	0.30909091	16	9
	sitosterol	16	0.00379603	0.31070496	16	10
Gene	NCOA2	55	0.08709096	0.4	55	1
	PGR	50	0.06735542	0.34744526	50	2
	PTGS1	41	0.04754372	0.39865997	41	3
	HSP90AA1	31	0.03375188	0.3908046	31	4
	PRKACA	29	0.01802402	0.38202247	29	5
	NR3C2	26	0.00947988	0.26832018	26	6
	PRSS1	24	0.02436126	0.38449111	24	7
	PPARG	24	0.0081081	0.36335878	24	8
	AR	22	0.03031946	0.38699187	22	9
	CHRM1	21	0.00742196	0.32827586	21	10

BC = betweenness centrality, CC = closeness centrality, JSD = Jianshen decoction.

### 3.2. DEGs of OPF

**Figure 3.**
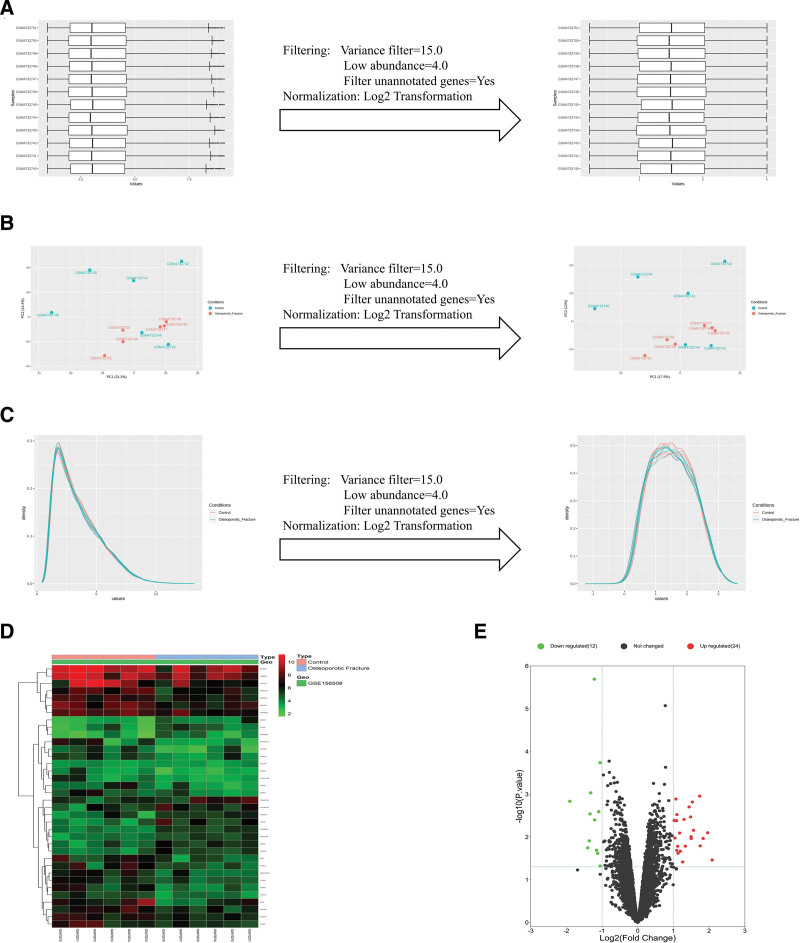
Bioinformatics analysis of GSE156508. (A) Box plots of samples before and after normalization. (B) PCA plot before and after normalization. (C) The plot of density against log2 of read counts. (D) Heat map of a total of 36 DEGs in GSE156508. (E) The volcano plot of a total of 36 DEGs in GSE156508. DEGs = differentially expressed genes, PCA = principal components analysis.

The quality assessment and quality control processing of the raw data was performed using FastQC software, and the Box plot, principal components analysis plot, and Density plot after data filtering and normalization are shown in Figure 3A–C. Using the DESeq2 package in the R language for differential analysis, 36 DEGs were screened, including 24 up-regulated genes and 12 down-regulated genes, and heat maps were drawn for the selected DEGs in GSE156508 (Fig. 3D), where red represents up-regulation of gene expression, and the green represents down-regulation of gene expression. Then, -log_10_ transformation was performed on the *P* values of genes processed by the analysis of variance. The DEGs were classified into up-regulated gene groups, down-regulated gene groups, and gene groups with no statistical differences according to log_2_ FC and -log_10_ (*P* value). The results were imported into the bioinformatics tool to draw volcano maps (Fig. 3E).

### 3.3. Core target genes of active ingredients of JSD acted on OPF

**Figure 4.**
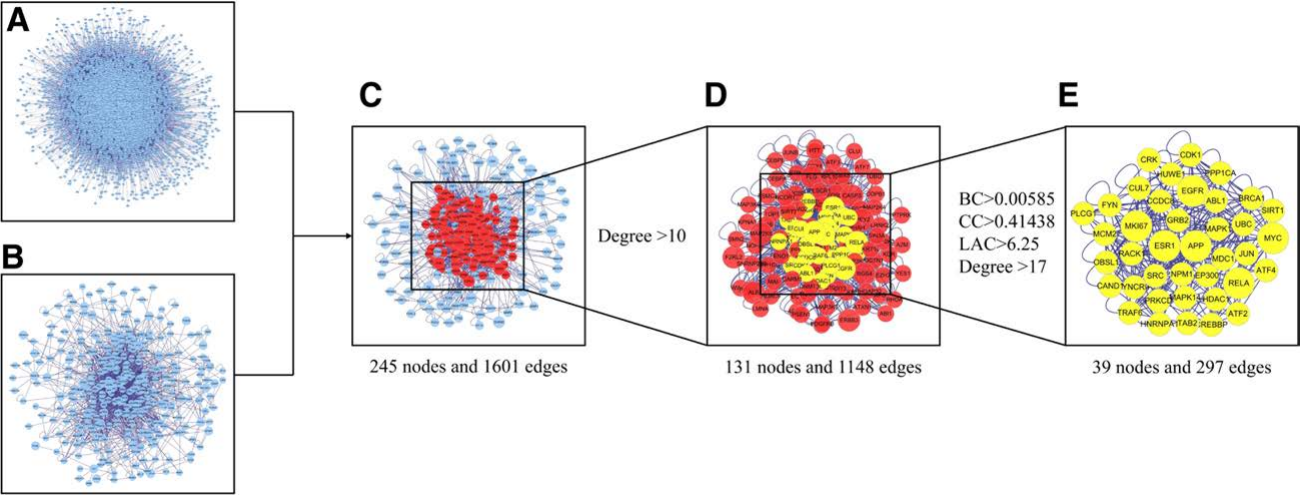
Process of topological screening for the PPI network. (A) PPI network of targets of JSD. (B) PPI network of DEGs of OPF. (C) PPI network of key targets of merging mapping. (D) PPI network of key genes with a screening threshold of degree > 10 (2 times the median value of degree). (E) PPI network of key genes with a second screening threshold of degree > 17, BC > 0.00585, CC > 0.41438, and LAC > 6.25 (the median value of all the above). BC = betweenness centrality, CC = closeness centrality, DEGs = differentially expressed genes, JSD = Jianshen decoction, LAC = local average connectivity, PPI = protein-protein interaction.

The PPI network of targets of JSD and the PPI network of DEGs of OPF were constructed respectively (Fig. 4A and B). These 2 PPI networks generated were intersected to obtain the PPI network shared by the targets of JSD and DEGs of OPF (Fig. 4C). The 131 potential target genes of the active ingredients of JSD acting on OPF were derived based on the 2-fold median values of Degree (Fig. 4D). The 39 potential core target genes of the active ingredients of JSD acting on OPF were derived based on the median values of Degree, betweenness centrality, closeness centrality, and median values of local average connectivity values (Fig. 4E).

### 3.4. PPI network of core target genes

**Figure 5.**
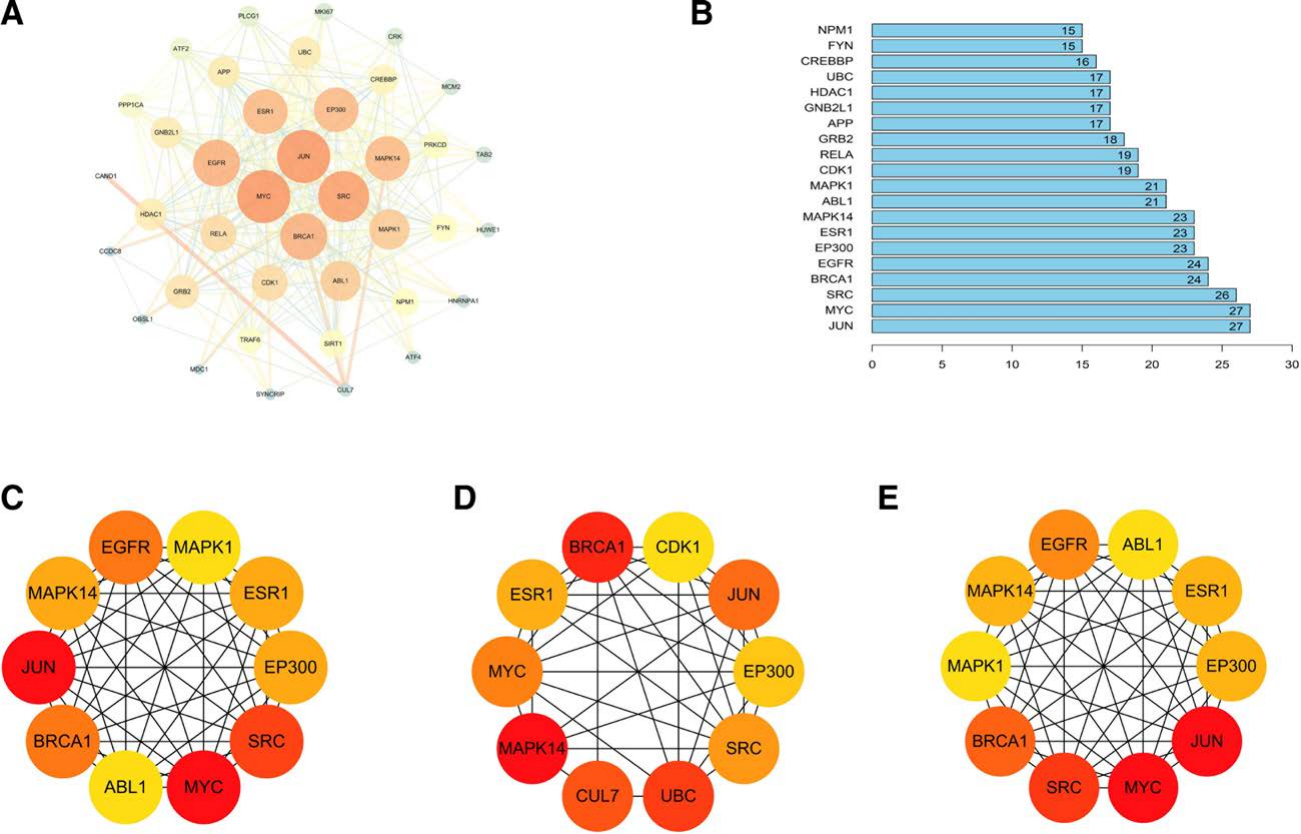
Analysis and mutual PPI network construction of potential core target genes. (A) PPI network generated on JSD-OPF mapped targets. (B) The top 20 hub genes according to the node degree. (C) PPI network of the top 10 hub genes based on Degree. (D) PPI network of the top 10 hub genes based on Betweenness. (E) PPI network of the top 10 hub genes based on Closeness. JSD = Jianshen decoction, OPF = osteoporotic fracture, PPI = protein-protein interaction.

The PPI network of core target genes of active ingredients of JSD acted on OPF was constructed by applying the STRING online analysis website (Fig. 5A), and the top 20 hub genes were screened according to node degree (Fig. 5B). The PPI networks of the top 10 hub genes based on Degree, Betweenness, and Closeness were constructed separately (Fig. 5C–E).

### 3.5. Enrichment analysis of GO/KEGG

**Figure 6.**
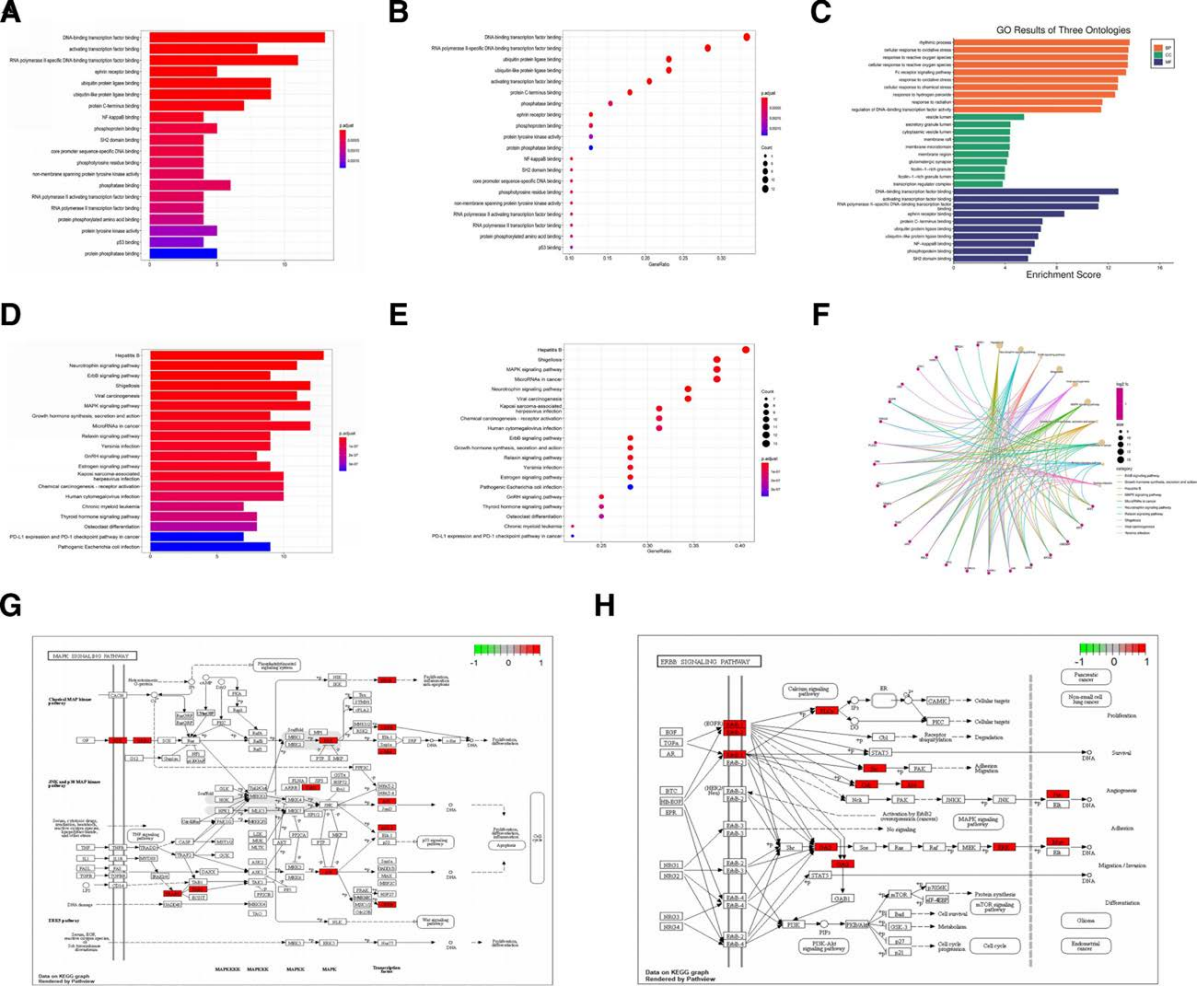
GO and KEGG enrichment analyses of 39 potential core target genes. (A) The Bar chart of the top 20 terms extracted according to the *P*.adjust value based on GO enrichment analysis. (B) The Bubble chart of the top 20 terms extracted according to the *P*.adjust value based on GO enrichment analysis. (C) Top 10 significantly enriched BP, CC, and MF categories based on gene ontology. (D) The Bar graph of the top 20 pathways extracted according to the *P*.adjust value based on KEGG enrichment analysis. (E) The Bubble chart of the top 20 pathways extracted according to the *P*.adjust value based on KEGG enrichment analysis. (F) Pathway cnetplot on KEGG enrichment analysis. (G) MAPK signaling pathway map. (H) ErbB signaling pathway map. BP = biological process, CC = closeness centrality, GO = gene ontology, KEGG = Kyoto encylopaedia of genes and genomes, MF = molecular function.

The GO and KEGG pathway enrichment analysis of the 39 potential core target genes was performed. The results showed that the GO analysis was mainly enriched in DNA-binding transcription factor binding, RNA polymerase II-specific DNA-binding transcription factor binding, ubiquitin protein ligase binding, ubiquitin-like protein ligase binding, activating transcription factor binding, etc. Biological processes were mainly focused on rhythmic process, cellular response to oxidative stress, and response to reactive oxygen species; Cellular components were mainly concentrated in the vesicle lumen, secretory granule lumen, and cytoplasmic vesicle lumen; Molecular function was mainly expressed as DNA-binding transcription factor binding, activating transcription factor binding and RNA polymerase II-specific DNA-binding transcription factor binding (Fig. 6A–C); KEGG pathway analysis revealed that it was mainly concentrated in Hepatitis B, Shigellosis, MAPK signaling pathway, MicroRNAs in cancer, Neurotrophin signaling pathway, Viral carcinogenesis, Kaposi sarcoma-associated herpesvirus infection and ErbB signaling pathway (Fig. 6D–F). The pathview package showed the signaling pathway diagram associated with core target genes (Fig. 6G and H).

### 3.6. Molecular docking verification

**Figure 7.**
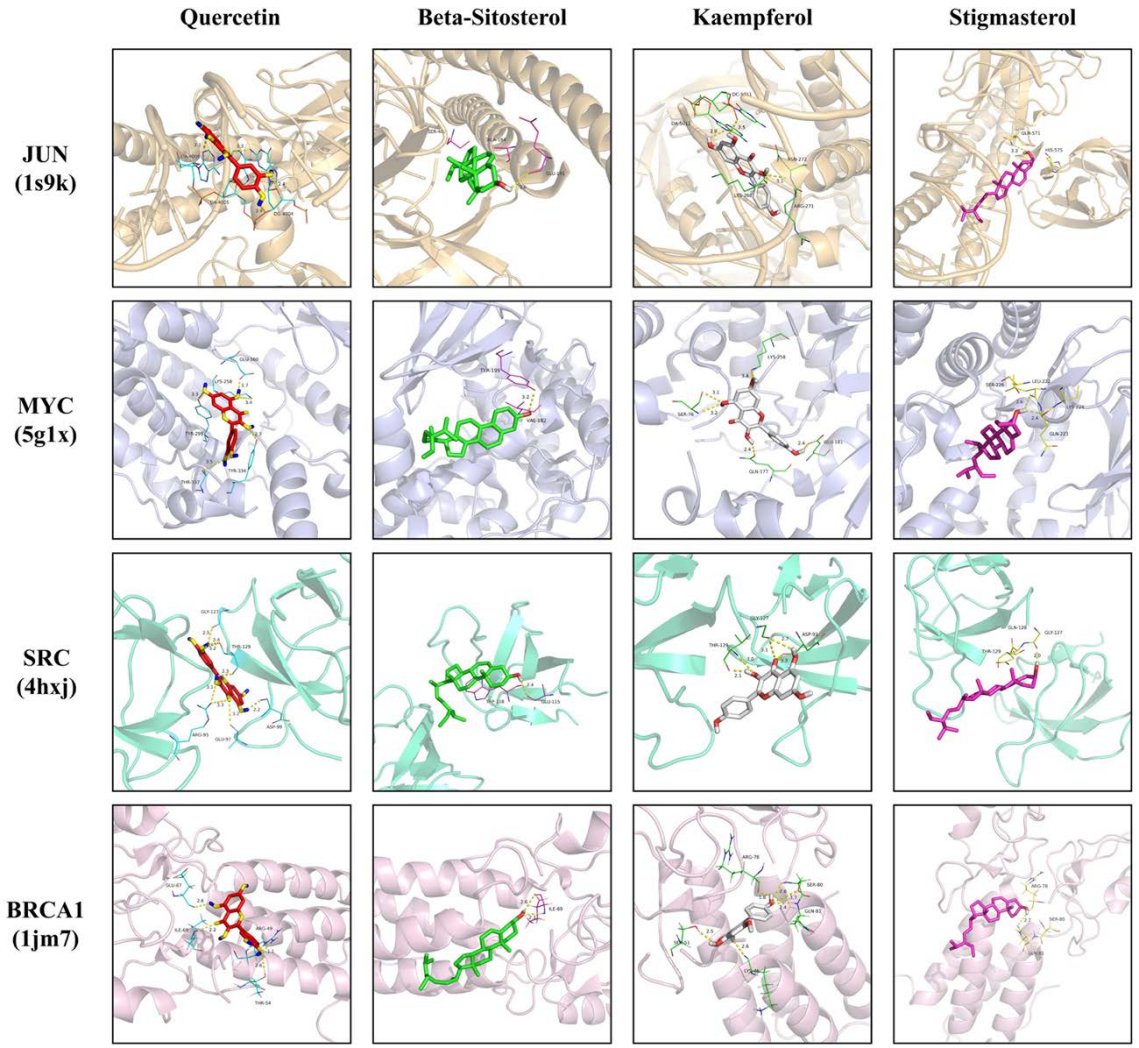
3D Molecular docking model of the 4 key active ingredients with the 4 hub targets.

**Figure 8.**
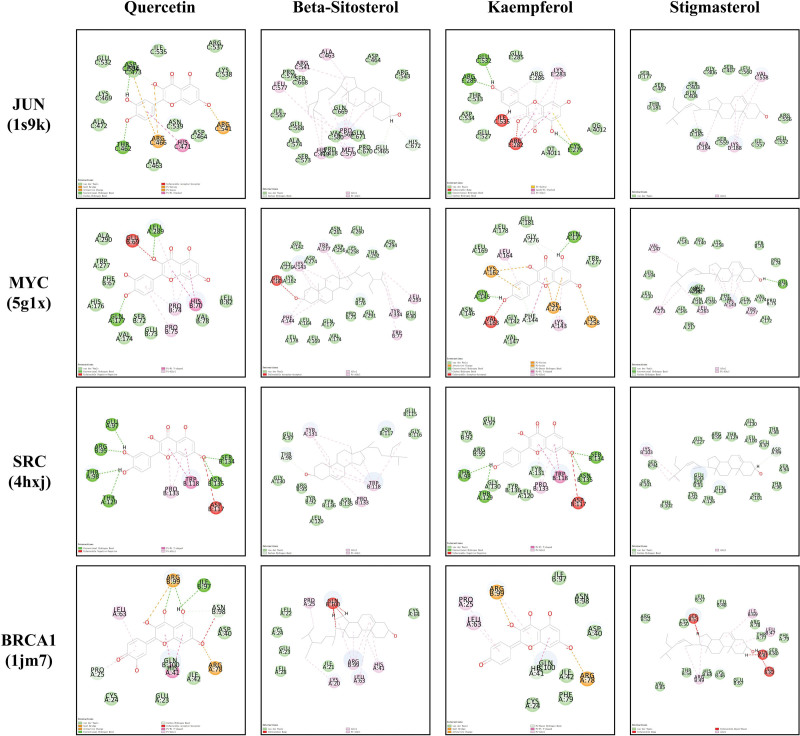
2D Molecular docking model of the 4 key active ingredients with the 4 hub-targets.

The top 4 active ingredients (Quercetin, Beta-sitosterol, Kaempferol, and Stigmasterol) were selected for screening and validated by molecular docking simulations with the related core proteins (JUN, MYC, SRC, and BRCA1) in that order, and the results showed that all active ingredients could form docking models with the core proteins in the docking results of the Vina script. In the LeDock software docking results, the binding energies of Beta-sitosterol with JUN, BRCA1, and Stigmasterol with SRC, and BRCA1 were greater than −5 kcal-mol^−1^, while all other combinations were able to form docking models. In addition, the docking results of DS 2019 software showed that Quercetin with MYC, SRC, Beta-sitosterol with JUN, and Stigmasterol with JUN, and MYC all formed stable docking models (Table 3). Comprehensive analysis revealed that SRC and Quercetin could form the most stable docking model. Finally, different core proteins form different dockers with different active molecules because they form different hydrogen and hydrophobic bonds at different amino acid sites. We imported the results calculated by Vina software into Pymol software for 3-dimensional molecular docking with protein ligands (Fig. 7), and DS 2019 software for 2-dimensional molecular docking with protein ligands (Fig. 8).

**Table 3:**
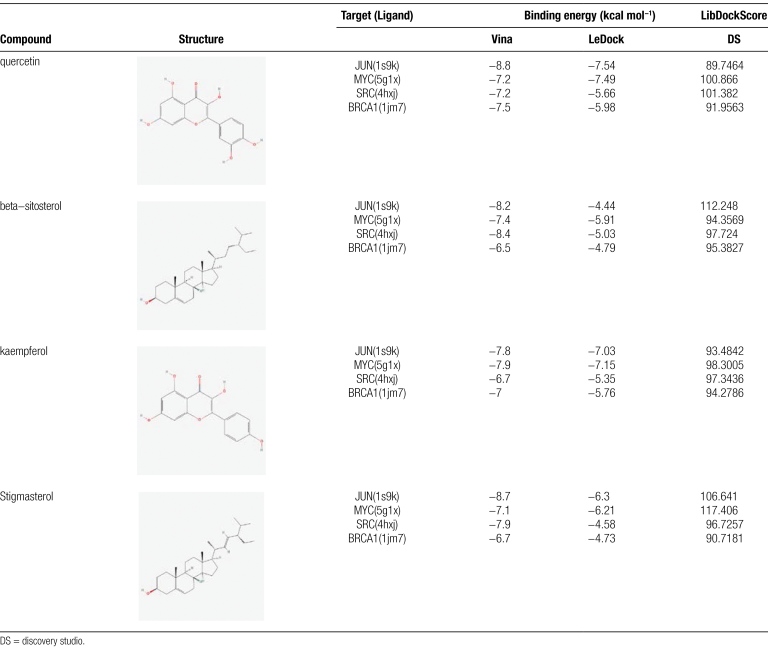
Comparison of the binding energy of molecular docking Vina, LeDock, and DS 2019.

### 3.7. Molecular dynamics simulation

**Figure 9.**
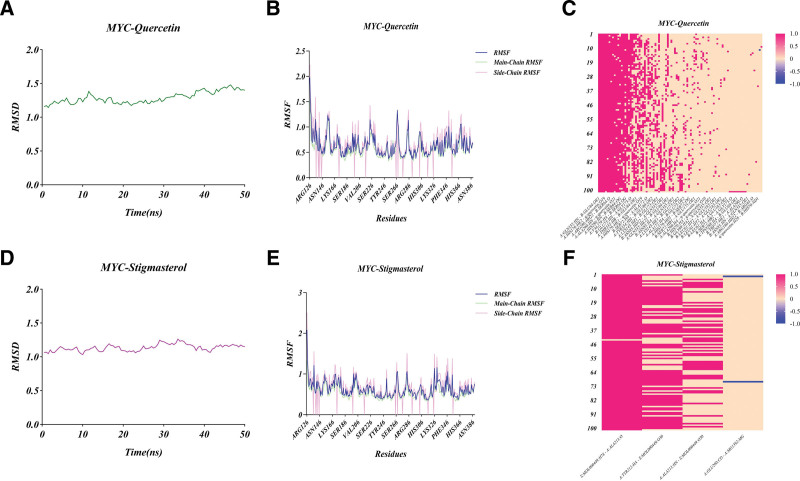
RMSD, RMSF, and hydrogen bonding heatmap of MD simulations. (A) RMSD of MYC-Quercetin. (B) RMSF of MYC-Quercetin. (C) hydrogen bonding heatmap of MYC-Quercetin. (D) RMSD of MYC-Stigmasterol. (E) RMSF of MYC-Stigmasterol. (F) hydrogen bonding heatmap of MYC-Stigmasterol. MD = molecular dynamics, RMSD = Root Mean Square Deviations, RMSF = root mean square fluctuations.

The optimal conformation resulting from the docking of MYC with Quercetin/Stigmasterol molecule was selected for subsequent molecular dynamics simulations and solved using an explicit periodic boundary solventized water model. In addition, for the analysis of the physiological environment, MYC-Quercetin complex was added with 711 water, 19 Sodium, and 25 Chloride, and MYC-Stigmasterol complex was added with 6495 water, 17 Sodium, and 30 Chloride. To investigate the structural stability of protein-ligand complexes during MD simulations, the RMSD values of the complexes were calculated for the complexes formed during docking for 50 ns during MD simulations (Fig. 9A and D), it can be seen that the complexes in both systems reached stability after 50 ns of MD simulation. In addition, the RMSD values of MYC-Quercetin complex mainly fluctuated between 1.14353 and 1.47945, and its mean RMSD value was 1.28998, and the RMSD values of MYC-Stigmasterol complex mainly fluctuated between 1.0329 and 1.26271, and its mean RMSD value was 1.13486. Both of these The RMSD fluctuation values of the 2 complexes were within the reasonable range, indicating that the structures of the complexes in the system were in equilibrium after the simulation, implying that MYC-Quercetin and MYC-Stigmasterol complexes were in a stable state during the whole MD simulation and played a stabilizing role in the formation of the complexes. In addition, the mean RMSD values of both complexes were <2.0, indicating that the 2 complexes were bound very stably. To analyze the fluctuation of various amino acids in the complex during the MD simulation, the root mean square fluctuations values of all amino acids during the simulation were calculated (Fig. 9B and E). The results showed that the MYC-Quercetin complex fluctuated more around amino acids GLN127, ILE135, SER155, GLN223, ALA267, and ARG285, and the MYC-Stigmasterol complex fluctuated more around amino acids GLN127, ARG137, ALA203, ALA267, LYS326, GLU330, while the remaining amino acids in the complex fluctuated less, which played a role in the stability of the complex. The hydrogen bonding heatmap during the simulations were shown in Figures 9C and F, from which it could be obtained that hydrogen bonding interactions were present in all conformations (especially in the red columns), indicating that these hydrogen bonds were very persistent and stable.

## 4. Discussion

JSD has a great safety profile, has been found to considerably enhance OP patients’ bone density, significantly reduce their pain, and boost serum E2, osteoprotegerin, and IGF-I levels^[13,14]^; however, the detailed underlying effect mechanisms remain unclear. This study aimed to combine bioinformatics, and computer virtual validation to explore the pharmacological mechanism of JSD for the treatment of OPF and to provide a basis for further research and clinical application of JSD.

In this study, we searched several powerful online databases to identify potential targets of JSD for the treatment of OPF. Through searching as well as screening, we obtained 70 potentially active ingredients and further obtained core compounds including Quercetin, Beta-sitosterol, Kaempferol, Stigmasterol, etc by analyzing the Degree values of active ingredient nodes and target gene nodes in the network. Quercetin is a flavonoid found in large quantities in fruits and vegetables. It has a wide range of biological activities. Animal studies have found that the effects of quercetin on bone are largely protective, with a few studies reporting negative results. Quercetin stimulates osteogenesis, angiogenesis, antioxidant expression, adipocyte apoptosis, and osteoclast apoptosis while inhibiting RANKL-mediated osteoclastogenesis, osteoblast apoptosis, oxidative stress, and inflammatory responses. Wnt, NF-B, Nrf2, SMAD dependency, and modulation of intrinsic and extrinsic apoptotic pathways are examples of potential processes that might be at play. Quercetin, on the other hand, has complicated and conflicting actions on the MAPK signaling pathway to control bone metabolism, leading to both stimulatory and inhibitory effects on bone.^[36]^ Quercetin also reduces ovariectomy-induced OP via controlling autophagy and apoptosis in rat osteoblasts.^[37]^ A phytosterol known as beta-sitosterol has been implicated in research as an anti-inflammatory, angiogenic, and hypocholesterolemic agent.^[38]^ According to research by Ruangsuriya et al, deleting beta-sitosterol and enriching quercetin and rutin increased the expression of osteogenic markers while decreasing the expression of osteolytic indicators.^[39]^ Kaempferol is a dietary bioflavonoid that is widely distributed in many plants and has several health advantages, such as antioxidant, anti-inflammatory, and anti-osteoporotic effects. Numerous studies using both in vitro and in vivo experimental models have validated the osteoprotective effects of kaempferol and plants containing it.^[40,41]^ In methylprednisolone-induced OP rats, Adhikary et al demonstrated that Kaempferol improved BMD, bone strength, and genes linked to bone synthesis while lowering genes linked to bone resorption. Additional effects of kaempferol include an increase in osteogenic markers and quicker bone repair at the fracture site.^[42]^ Endochondral and intramembranous ossification combine to make bone. MSCs undergo chondrocyte differentiation during endochondral ossification, generating the cartilage matrix, which is thereafter gradually replaced by bone. MSCs are directly differentiated into osteoblasts during the process of intramembranous ossification.^[43]^ OP advances as a result of MSCs’ propensity to develop into adipocytes rather than osteoblasts and chondrocytes.^[44–46]^ Kaempferol encourages MSCs to differentiate into osteoblasts by upregulating the expression of ALP, Runx-2, OSX, and OCN, while it prevents MSCs from diffusing into adipocytes by downregulating PPAR-γ.^[47]^ Stigmasterol is a phytosterol or plant lipid. Previous research has shown that phytosterols can be employed as therapeutic mediators, such as antipyretic, anticancer, and immunomodulatory agents for several disorders.^[48–50]^ Stigmasterol has an anti-inflammatory mechanism that involves decreasing the expression of NF-kBp65 and p38MAPK in joints, which decreases the expression of pro-inflammatory mediators and enhances the expression of anti-inflammatory cytokines.^[51]^ Our work showed that JSD was effective in the treatment of OPF through the combination of multiple components.

Subsequently, by PPI network analysis, we identified 39 core genes associated with the JSD treatment of OPF, including JUN, MYC, SRC, BRCA1, MAPK14, MAPK1, ESR1, and EP300. Immediately after, we performed GO and KEGG pathway enrichment analysis on these 39 core genes, and the results showed that “DNA-binding transcription factor binding,” “RNA polymerase II- specific DNA-binding transcription factor binding,” “ubiquitin protein ligase binding,” and “MAPK signaling pathway.” "MAPK signaling pathway” and “ErbB signaling pathway” may be potential pathways for JSD treatment of OPF. Among them, JUN, MYC, and MAPK1 are all involved in regulating the MAPK signaling pathway and the ErbB signaling pathway. The MAPK includes the ERKs, p38, and c-Jun N-terminal kinases.^[52]^ The biological activities ERK, c-Jun N-terminal kinase, and p38 MAPKs are engaged in include cell survival, proliferation, and transformation.^[53]^ It was demonstrated that Achyranthes bidentata saponins might stimulate bone marrow stromal cell proliferation and osteoblast development by activating the ERK signaling pathway.^[54]^ Inflammation, bone scab development, and remodeling are the 3 steps that generally correspond to the complicated biological process of fracture healing. The effectiveness of fracture healing depends on how well these processes are coordinated. However, in OPFs, recruitment of reparative mesenchymal stem cells, neoangiogenesis of healing tissue, levels of endochondral ossification, healing tissue remodeling rate, and estrogen-expressing receptors are compromised, resulting in delayed healing or worse healing results.^[55–59]^ Inhibition of p38 MAPK decreased in vivo production of inflammatory cytokines, callus development, and acute inflammatory response.^[60]^ The ErbB family of receptor tyrosine kinases consists of ErbB1, ErbB2, ErbB3, ErbB4, and multiple ligands including epidermal growth factor, transforming growth factor-α, and others.^[61–63]^ ErbB signaling has a role in the timely maturation of chondrocytes and the differentiation of periosteal osteoblasts.^[64]^ Activation of EGFR has been reported to increase the number of osteoprogenitor cells by promoting cell proliferation and inhibiting serum depletion-induced or TNF-α-induced apoptosis.^[65]^ In mice, suppression of EGFR in bone progenitors and osteoblasts or inactivation of EGFR by EGFR-specific inhibitors causes bone loss as a result of a decrease in bone marrow mesenchymal progenitor cells.^[66]^ On the endosteal surface, EGFR in osteoprogenitor cells prevented cellular senescence and repressed cortical bone deterioration. The survival of osteoprogenitor cells in bone depends on EGFR signaling. Additionally, Liu et al showed that blocking the ERK/MAPK pathway prevented osteoprogenitor cells produced by EGF treatment from surviving and proliferating and instead accelerated the senescence of osteoprogenitor cells in bone.^[67]^ This is consistent with previous reports that EGFR signaling promotes the proliferation of hair follicle-derived MSCs through ERK1/2 and suppresses p16INK4a expression.^[68]^ In the present study, our bioinformatics analysis showed that JSD can play a therapeutic role in OPF by regulating the ERK/p38/MAPK signaling pathway as well as the ErbB1/ErbB signaling pathway.

Finally, to further validate the binding ability of JSD core active ingredients to molecular target domains, we validated the screening of the top 4 active ingredients and target proteins by molecular docking technique. The results showed that: Quercetin and kaempferol could form stable docking models with JUN, MYC, SRC, and BRCA1, respectively. Beta-sitosterol could dock with MYC and SRC protein ligands. And Stigmasterol could form stable docking with JUN and MYC protein ligands. Through the computational analysis of Vina, LeDock, and DS software, we concluded that Quercetin/Stigmasterol could form a very stable docking model with the 5g1x ligand of MYC protein. Immediately afterward, to further investigate the binding process between the core protein and the active ingredient, we performed molecular dynamics simulations of MYC-quercetin and MYC-Stigmasterol complexes, and the results of the molecular dynamics simulations further justified the results of molecular docking. MYC (also known as c-MYC) is a broad-ranging transcription factor that regulates cell differentiation and proliferation programs through a variety of mechanisms. In the RANKL-induced transcription program, MYC is a crucial transcription factor and is connected to osteoclastogenesis in vitro.^[69]^ In this study, we found that MYC in combination with Quercetin and Stigmasterol of JSD exerted anti-OPF effects.

There are however several limitations of this study. First, the results of this study lack cellular and animal experimental validation. Another limitation is the false negative due to targets from different databases, which may have biased effects due to different experimental conditions.

## 5. Conclusions

In summary, the active ingredients of JSD and their molecular targets in OPF were successfully revealed through bioinformatics, molecular docking, and molecular dynamics simulations. We identified a total of 70 potential key components and 39 core targets. JSD was found to play a role in the treatment of OPF possibly through the regulation of MAPK and ErbB signaling pathways. The interaction between Quercetin/Stigmasterol and MYC is important for the mechanism of OPF treatment by JSD, but further in vivo/in vitro trials are needed. This study provided an overall view of the potential pharmacological mechanisms of JSD against OPF, however, there was no corresponding experimental validation of this study, which is something we need to do in the future.

## Acknowledgments

Grateful Acknowledgments is made to my supervisor Professor Huo Liwei and Wang haibin who gave me considerable help means of suggestions, comments, and criticism. In addition, I deeply appreciate the contribution to this thesis made in various ways by my friends and classmates.

## Author contributions

**Conceptualization:** Weinian Liu.

**Data curation:** Weinian Liu, Mengting Hu.

**Formal analysis:** Weinian Liu, Yuanhao Hu.

**Methodology:** Weinian Liu, Weijian Chen, Guangwei Wang.

**Software:** Weinian Liu, Yidong Xu, Jun Tan.

**Supervision:** Weinian Liu, Guangwei Wang, Qi He, Haibin Wang, Liwei Huo.

**Validation:** Weinian Liu, Weijian Chen, Mengting Hu, Qi He, Yidong Xu, Jun Tan.

**Visualization:** Weinian Liu, Weijian Chen, Yuanhao Hu.

**Writing – original draft:** Weinian Liu.

**Writing – review & editing:** Weinian Liu, Haibin Wang, Liwei Huo.
